# Influenza‐specific IgG1^+^ memory B‐cell numbers increase upon booster vaccination in healthy adults but not in patients with predominantly antibody deficiency

**DOI:** 10.1002/cti2.1199

**Published:** 2020-10-16

**Authors:** Gemma E Hartley, Emily S J Edwards, Julian J Bosco, Samar Ojaimi, Robert G Stirling, Paul U Cameron, Katie Flanagan, Magdalena Plebanski, Philip Mark Hogarth, Robyn E O’Hehir, Menno C van Zelm

**Affiliations:** ^1^ Department of Immunology and Pathology Central Clinical School Monash University Melbourne VIC Australia; ^2^ The Jeffrey Modell Diagnostic and Research Centre for Primary Immunodeficiencies Melbourne VIC Australia; ^3^ Department of Allergy, Immunology and Respiratory Medicine Central Clinical School Alfred Hospital Monash University and Allergy, Asthma and Clinical Immunology Service Melbourne VIC Australia; ^4^ Infectious Diseases Monash Health Clayton VIC Australia; ^5^ Immunology Laboratory Monash Pathology Clayton VIC Australia; ^6^ Allergy and Immunology Monash Health Clayton VIC Australia; ^7^ School of Medicine University of Tasmania Launceston TAS Australia; ^8^ School of Health and Biomedical Sciences RMIT Bundoora VIC Australia; ^9^ Immune Therapies Group Burnet Institute Melbourne VIC Australia

**Keywords:** antigen‐specific memory B cells, influenza, predominantly antibody deficiency, vaccination

## Abstract

**Background:**

Annual influenza vaccination is recommended to all individuals over 6 months of age, including predominantly antibody deficiency (PAD) patients. Vaccination responses are typically evaluated by serology, and because PAD patients are by definition impaired in generating IgG and receive immunoglobulin replacement therapy (IgRT), it remains unclear whether they can mount an antigen‐specific response.

**Objective:**

To quantify and characterise the antigen‐specific memory B (Bmem) cell compartment in healthy controls and PAD patients following an influenza booster vaccination.

**Methods:**

Recombinant hemagglutinin (HA) from the A/Michigan/2015 H1N1 (AM15) strain with an AviTag was generated in a mammalian cell line, and following targeted biotinylation, was tetramerised with BUV395 or BUV737 streptavidin conjugates. Multicolour flow cytometry was applied on blood samples before and 28 days after booster influenza vaccination in 16 healthy controls and five PAD patients with circulating Bmem cells.

**Results:**

Recombinant HA tetramers were specifically recognised by 0.5–1% of B cells in previously vaccinated healthy adults. HA‐specific Bmem cell numbers were significantly increased following booster vaccination and predominantly expressed IgG1. Similarly, PAD patients carried HA‐specific Bmem cells, predominantly expressing IgG1. However, these numbers were lower than in controls and did not increase following booster vaccination.

**Conclusion:**

We have successfully identified AM15‐specific Bmem cells in healthy controls and PAD patients. The presence of antigen‐specific Bmem cells could offer an additional diagnostic tool to aid in the clinical diagnosis of PAD. Furthermore, alterations in the number or immunophenotype of HA‐specific Bmem cells post‐booster vaccination could assist in the evaluation of immune responses in individuals receiving IgRT.

## Graphical abstract

We here developed an extensive flowcytometry panel to examine influenza‐specific memory B cells using fluorescently labeled recombinant haemagglutinin (HA) tetramers. Influenza‐specific memory B cells were predominantly IgG1^+^, and these were increased following booster vaccination in healthy controls, but not in patients with predominantly antibody deficiency.

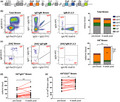

## Introduction

Predominantly antibody deficiency (PAD) comprises the largest group of patients (~70%) with a primary immunodeficiency disorder (PID).^1^, ^2^, ^3^, ^4^, ^5^ Patients are defined by an impaired antibody (Ab) response to antigen stimulation.^6^, ^7^, ^8^ Within the group of PAD patients, those with a complete absence of serum immunoglobulin (Ig) and of circulating B cells are defined as having agammaglobulinemia. This subgroup is genetically well defined with the majority being males having X‐linked inheritance because of mutations in the *BTK* gene,^9^, ^10^ and others having autosomal recessive inheritance because of mutations in pre‐B‐cell receptor signalling molecules or critical transcription factors.^11^, ^12^, ^13^, ^14^, ^15^, ^16^, ^17^ Other forms of PAD are less well defined genetically and diagnosed based on impaired vaccination responses and varying degrees of reductions of Ig isotypes in serum. Common variable immunodeficiency (CVID) is defined by reduced serum IgG in conjunction with reduced IgA and/or IgM.^6^, ^18^, ^19^, ^20^ Patients with hypogammaglobulinemia (HGG) have reduced IgG in the context of normal IgA and IgM.^6^, ^18^, ^20^ Finally, patients with impaired vaccination responses in the context of normal total IgG distinguish those with IgG subclass deficiency and those with specific Ab deficiency.^5^, ^6^, ^20^, ^21^

Typically, treatment of PAD involves Ig replacement therapy (IgRT) and prophylactic antibiotics to reduce the number and severity of infections.^7^, ^21^, ^22^ This is important to reduce the risk of irreversible organ damage, such as bronchiectasis because of recurrent respiratory infections that is a major contributor to the high morbidity and reduced life expectancy of patients.^7^, ^22^, ^23^ PAD patients can also develop non‐infectious complications such as autoimmunity and malignancy.^6^, ^7^, ^21^, ^22^, ^24^ These complications are difficult to treat and, if prominent, can mask a PAD diagnosis resulting in a significant delay in diagnosis and initiation of optimal treatment.^7^, ^23^

With the diagnostic rate of genomics in PAD being only ~ 20%,^5^, ^7^, ^25^, ^26^, ^27^ immunophenotyping of the blood B‐cell compartment is frequently performed to support the clinical diagnosis.^6^, ^28^, ^29^ Typically, PAD patients have strongly reduced memory B (Bmem) cell numbers and these involve both the ‘unswitched’ CD27^+^IgM^+^IgD^+^ as well as ‘Ig class switched’ CD27^+^IgM^−^IgD^−^ subset that includes IgG and IgA Bmem cells. Although reduced within the PAD group, many patients have detectable Bmem cell numbers, and in some patients, these are even in the normal range of age‐matched controls.^5^, ^6^, ^18^, ^30^ The presence of Bmem cells is associated with fewer complications (especially bronchiectasis and enteropathy),^5^, ^23^ although it remains unclear whether these Bmem cells are fully functional.

Bmem cells are defined as being resting lymphocytes with a pre‐activated phenotype and molecular signs of having encountered antigen, that is somatic hypermutations (SHMs) in their Ig‐variable regions and/or Ig class switch recombination (CSR). In human blood, many subsets can be defined based on the expression of the various Ig isotypes and presence or absence of CD27 expression.^31^, ^32^, ^33^ Of these, CD27^+^IgM^+^IgD^−^ and CD27^−^IgG^+^ Bmem cells appear to be predominantly derived from primary germinal centre (GC) responses. CD27^+^IgG^+^ show evidence of secondary responses with a more extensive replication history,^31^ increased SHM and increased usage of IgG2, derived from indirect switching through IgG1.^31^, ^34^ Therefore, the examination of isotypes can be used to infer whether Bmem cells are likely derived from primary (IgG1 or IgG3) or secondary (IgG2 or IgG4) GC reactions. Using expression of CD71 as a marker of recent activation or fluorescently labelled antigen,^35^ it has been shown that Bmem cells are generated after 10‐14 days, that is after the initial wave of plasmablasts at ~ 7 days.

Although the main contribution of Bmem cells to functional immunity is thought to lie in their capacity to rapidly produce high‐affinity antibodies upon secondary encounter with the same pathogen,^31^ Bmem cells can also act as effective antigen‐presenting cells (APCs) to naive CD4^+^ T cells in the context of influenza vaccination.^36^ Thus, even if Bmem cells in PAD patients are incapable of differentiating into Ab‐producing plasma cells, antigen‐specific Bmem cells can support T‐cell immunity to viral pathogens. The influenza virus causes a respiratory infection,^37^, ^38^, ^39^ with a high risk of secondary infections by bacterial pathogens. In PAD patients, influenza can lead to hospitalisation and more serious respiratory conditions such as pneumonia,^40^, ^41^ and therefore, vaccination is recommended.^37^, ^41^, ^42^, ^43^ It is known that IgRT products contain high neutralising antibody levels to the influenza virus,^44^, ^45^ and hence, it is difficult to distinguish between a response generated by the patient and donated antibody. Additionally, healthy individuals who have been previously vaccinated possess high titres of neutralising antibodies,^46^, ^47^, ^48^ which make it difficult to measure a response in this manner.

In recent studies, recombinant forms of the hemagglutinin (HA) antigen of influenza have been successfully used to detect circulating Bmem cells in the blood of healthy controls between 14 and 28 days post‐vaccination using fluorescent antigen tetramers and flow cytometry.^35^, ^49^, ^50^, ^51^, ^52^, ^53^, ^54^ Here, we utilised this approach to examine presence of HA‐specific Bmem in previously vaccinated individuals, and the effect of the 2019 seasonal influenza vaccination as a boosted response to previous immunisation with the A/Michigan/45/2015 (H1N1)pdm09‐like (AM15). The numbers and immunophenotype of influenza‐specific Bmem cells were determined in healthy adults, as well as in patients with PAD.

## Results

### Production of recombinant HA protein and tetramers

**Figure 1:**
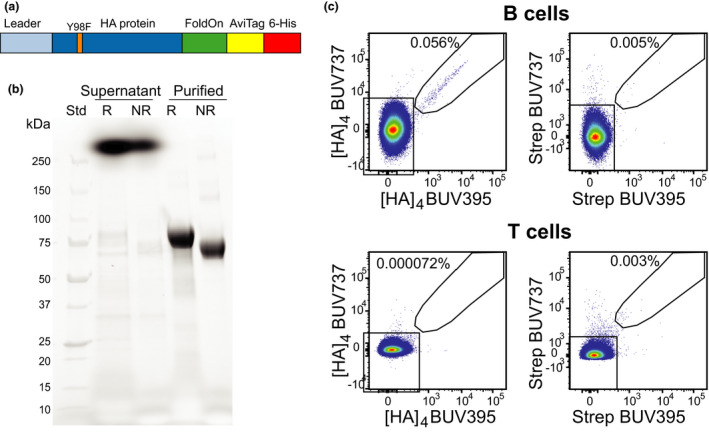
Design and production of recombinant AM15 HA protein and tetramers. **(a)** AM15 HA protein construct design. **(b)** Unstained gel of supernatant of AM15 transfection and purified protein. **(c)** Staining of BUV395/737 AM15 HA tetramers and BUV395/737 streptavidin on B cells and T cells. Percentages indicate the proportions of HA‐specific cells within total B or T cells. For gating strategy and population definitions, see Supplementary figure 1. AM15, A/Michigan/45/2015 (H1N1)pdm09‐like HA protein. Std, standard. R, reducing conditions. NR, non‐reducing conditions.

To detect influenza‐specific B cells, we generated recombinant HA protein with targeted modifications to facilitate tetramerisation of natively folded protein. HA from the AM15 strain was chosen as it was included in the 2017–2019 vaccines. The recombinant AM15 HA protein contained the previously reported Y98F mutation to prevent binding of the protein to sialic acids expressed on the cell surface of lymphocytes,^54^ as well as a trimeric FoldOn domain, AviTag and 6His for trimerisation, targeted biotinylation and purification, respectively (Figure 1a).^54^ The 64 kDa HA protein was successfully produced following transient transfection in Expi293F cells and purification from culture supernatant (Figure 1b). Following biotinylation, two tetramers of the HA protein were generated, one with streptavidin‐BUV395 and one with streptavidin‐BUV737, which were used concurrently to allow double discrimination of the HA‐specific B cells (Figure 1c). HA‐specific B cells were rare events at 0.5–1% of B cells, but staining was highly specific with < 0.01% of positive events detected using streptavidin‐only controls or T cells exposed to HA tetramers (Figure 1c).

### Booster vaccination increases circulating AM15‐specific memory B cells expressing IgG1

To examine the effect of influenza booster vaccination on the numbers and immunophenotype of Bmem cells, 16 healthy adults were recruited who had previously received the 2018 influenza vaccine and had decided to receive the 2019 vaccine. Blood samples from all adults were taken prior to and then approximately 28 days (range 26–33 days) after administration of the 2019 vaccine (Table 1). At baseline, 13 healthy controls (81%) were confirmed to have normal numbers of B cells and all healthy controls had normal numbers of T cells. All healthy controls had high neutralising antibody titres as determined with the hemagglutination inhibition (HI) assay (Table 1 **,** Supplementary table 1).

**Table 1:** Clinical and immunological features of healthy controls

Control	Age at booster (years)	Sex	Days post‐vaccination sample	T cells per μL blood	B cells per μL blood	Bmem per μL blood	HI titre^a^ pre‐booster
1	39	M	31	1072	169	47	640
2	29	F	33	976	151	30	640
3	35	F	31	2128	325	110	1280
4	33	F	28	1236	200	**24**	640
5	26	F	27	1558	172	61	320
6	39	F	32	842	**82**	36	640
7	24	M	28	1771	268	52	1280
8	25	F	31	1639	226	84	1280
9	45	F	28	1238	**82**	**16**	640
10	29	F	29	947	**71**	30	640
11	33	M	28	1231	119	25	640
12	39	F	26	1885	225	131	640
13	25	M	28	1451	126	51	640
14	24	M	28	1314	185	61	640
15	31	F	28	1503	182	57	1280
16	43	F	32	1341	115	59	640

^a^ A serum HI titre of ≥ 40 is assumed to indicate neutralising capacity.^46^ Values below normal range are depicted in bold. Reference range: B cells, 97–614 cells µL^–1^; T cells, 823–2491 cells µL^–1^; Bmem, 27–154 cells µL^–1^.^6^ For cell population definitions, see Supplementary figure 1. Bmem, memory B cells.

**Figure 2:**
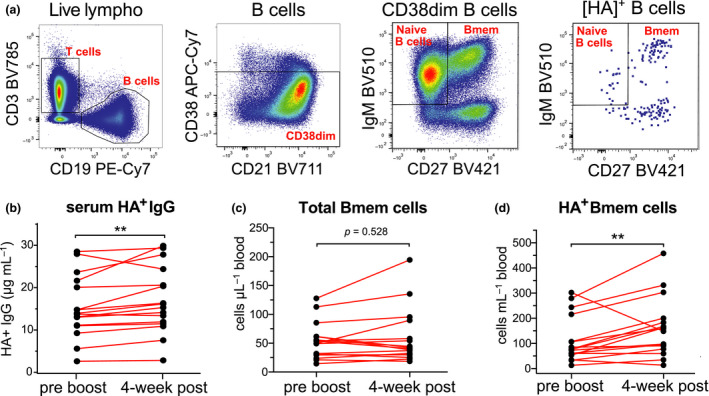
AM15 HA‐specific B memory cell numbers increase following booster vaccination in healthy controls. **(a)** Gating strategy to discriminate B cells from T cells, followed by selection of CD38^dim^ B cells in which naïve and memory subsets were distinguished using IgM and CD27. Similarly, within CD38^dim^ HA‐specific (HA^+^) B cells (Figure 1), naïve and memory subsets were defined. **(b)** Serum HA‐specific IgG levels pre‐ and post‐booster vaccination as measured by ELISA. **(c)** Total memory B (Bmem) cell numbers pre‐ and post‐booster vaccination. **(d)** HA^+^ Bmem cell numbers pre‐ and post‐booster. Statistics were performed with the Wilcoxon matched‐pairs signed rank test; ** *P* < 0.01.

To determine the absolute number and immunophenotype of HA‐specific Bmem cells, we stained PBMC from the healthy controls with the AM15 HA tetramers together with monoclonal antibodies (mAbs) to immunophenotype Bmem cells (Figure 2; Supplementary table 2). An average of 221 HA‐specific events were acquired (range 74–512 events) to allow for the characterisation of HA‐specific Bmem into IgG subclasses. The HA‐specific Bmem cells were defined as being double‐positive for the HA tetramers, being CD19^+^CD38^dim^ and following exclusion of CD27^−^IgM^+^ naive B cells (Figure 2a; Supplementary figure 1a).^6^, ^31^, ^34^

**Figure 3:**
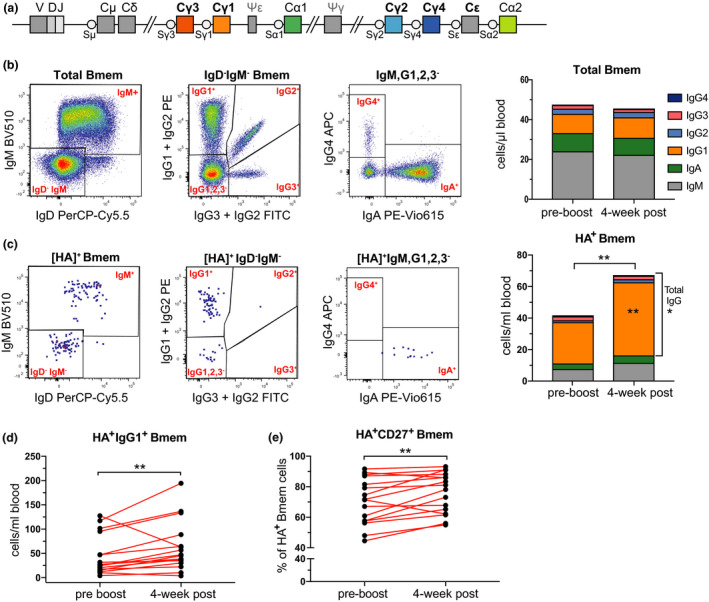
Predominant IgG1 response in AM15 HA‐specific B cell memory in healthy controls. **(a)** Schematic of the constant gene regions in the human *IGH* locus depicting their relative positioning with respect to the rearranged VDJ exon encoding the variable domain. **(b)** Gating strategy to delineate IgM^+^, IgG1^+^, IgG2^+^, IgG3^+^, IgG4^+^ and IgA^+^ CD19^+^ CD38^dim^ memory B cells (Bmem) and their median cell numbers pre‐ and post‐vaccination. **(c)** Gating strategy to delineate IgM^+^, IgG1^+^, IgG2^+^, IgG3^+^, IgG4^+^ and IgA^+^ CD19^+^ CD38^dim^ HA‐specific (HA^+^) Bmem and their median cell numbers pre‐ and post‐vaccination. **(d)** HA^+^ IgG1^+^ Bmem cell numbers pre‐ and post‐vaccination **(e)** Frequencies of HA^+^ Bmem cells expressing CD27 pre‐ and post‐booster vaccination. Statistics were performed with the Wilcoxon matched‐pairs signed rank test; * *P* < 0.05, ** *P* < 0.01.

Consistent with the fact that all individuals had previously been vaccinated, they all had substantial levels of AM15 HA‐specific IgG in their serum (Figure 2b). These levels were consistently increased (*P* < 0.01) post‐vaccination, although the extent was limited (average, 1.1‐fold change; Figure 2b). Total Bmem cell numbers did not change following booster vaccination (Figure 2c). HA‐specific Bmem cells were readily detectable both pre‐ and post‐booster vaccination in all 16 healthy controls, and their numbers were significantly increased post‐booster vaccination (*P* = 0.0092; Figure 2d). Total and HA‐specific Bmem cells were further immunophenotyped to determine preferential usage of the distinct isotypes (IgA, IgG or IgM) and IgG subclasses (IgG1, IgG2, IgG3 and IgG4) encoded by the *IGH* locus (Figure 3a) in these individuals (Figure 3b and c, Supplementary figure 1b,c). Within the total Bmem compartment, about half of the cells express IgM, followed by about 20% of both IgA and IgG1 expressing cells, and within the remaining 10%, the majority expressed IgG2 and only few IgG3^+^ and IgG4^+^ cells (Figure 3b; Supplementary figure 2). In contrast, HA‐specific Bmem cells predominantly expressed IgG1 and these consisted of ~ 2/3 of the population (Figure 3c). We observed a significant increase in the number of HA‐specific IgG1^+^ Bmem cells post‐booster (*P* = 0.0092; Figure 3d). Numbers of HA‐specific IgM, IgA, IgG2, IgG3 or IgG4 Bmem cells were not significantly changed post‐vaccination (*P *> 0.05; Figure 3b). The majority of HA‐specific IgG1^+^ Bmem cells were CD27^+^, and as a result, there was an increase in the frequency of CD27^+^ HA‐specific Bmem cells within the total HA‐specific memory population (Figure 3e).

### Clinical and immunological features of antibody‐deficient patients

To study the effect of influenza booster vaccination on antigen‐specific Bmem cells in PAD, we examined five PAD patients, who were enrolled in our research study at the Alfred Hospital and Monash Health in Melbourne, Australia.^6^, ^55^ The five patients were selected on the basis of having numbers of B cells and Bmem cells within the normal range (Table 2), having previously received the 2018 influenza vaccination, and had expressed their plan to receive the 2019 vaccine of their own volition. The median age of the patients was 41 years (range 29–50), and the majority (4/5) were females (Table 2). Three patients were diagnosed with CVID, one with HGG and one with an unclassified Ab deficiency on the basis of clinical characteristics and serum Ig levels (Table 2). To date, none of the five patients had been genetically diagnosed. All patients presented with recurrent infectious respiratory complications including sinusitis, otitis media and pneumonia. In two patients (40%), this was accompanied by non‐infectious complications (autoimmunity; Table 2). Four out of the five patients were receiving IgRT at the time of inclusion, and all patients had high HI neutralising titres pre‐booster vaccination (Table 2, Supplementary table 1).

**Table 2:** Clinical and immunological features of the patients with predominantly antibody deficiency

Patient	Age at booster vaccination	Sex	Sampling post‐booster	Clinical Diagnosis	T cells	B cells	Bmem	IgG	IgA	IgM	Infectious complications	Non‐infectious complications	IgRT at time of sampling	HI titre pre‐boost	Patient classification		Verstegen *et al*. 2019 ^55^	Edwards *et al*. 2019 ^6^
	(years)		(days)		cells μL^–1^	cells μL^–1^	cells μL^–1^	(g L^–1^)	(g L^–1^)	(g L^–1^)					Freiburg^73^	EUROclass^74^		
1	50	F	29	CVID	**818**	180	29	**3**	**0.5**	0.44	otitis, sinusitis, shingles	Urticaria	YES	320	II	B + smB+ CD21norm Trnorm	‐	28
2	32	F	29	HGG	1982	144	34	**5.2**	**0.3**	0.5	asthma	none	YES	320	II	B + smB+ CD21loTrnorm	PAD‐08	44
3	29	M	29	CVID	**3355**	467	30	**5.6**	**0.6**	**0.3**	sinusitis	none	YES	640	II	B + smB+ CD21norm Trnorm	PAD‐06	17
4	27	F	56–91	CVID	2008	166	43	**3.9**	**0.7**	**0.3**	Pneumonia, bronchitis	Vitiligo	YES	320	II	B + smB+ CD21normTrhi	‐	18
5	49	F	62	Unclassified^a^	833	144	30	**5.2**	1.2	1.5	sinopulmonary, recurrent HSV, shingles	None	No	640	II	B + smB+ CD21norm Trnorm	‐	‐
Normal range					823–2491	97–614	27–154	6.10–16.2	0.85–4.99	0.35–2.42								

Values below normal range are depicted in bold font and above normal range underlined. Reference ranges: Edwards *et al*. 2019.^6^ For population definitions, see Supplementary figure 1.

CVID, common variable immunodeficiency; HGG, hypogammaglobulinemia; smB, switched memory B cells; Tr, transitional B cells; Bmem, memory B cells.

Freiburg classification definitions: smB^−^, <0.4% CD27^+^ IgD^−^ B cells within lymphocytes; CD21lo, ≥ 20% B cells are CD21lo.^73^

EUROclass definitions: B^−^ < 1% of lymphocytes; smB^−^, < 2% of B cells; CD21lo, ≥ 10% B cells are CD21lo; Trhi,> 9% of B cells are transitional (CD38^hi^ CD27^−^).^74^

^a^ Patient has reduced total IgG, with poor response to polysaccharide pneumococcal vaccination (4/15 serotypes).

### PAD patients can generate HA‐specific Bmem cells

**Figure 4:**
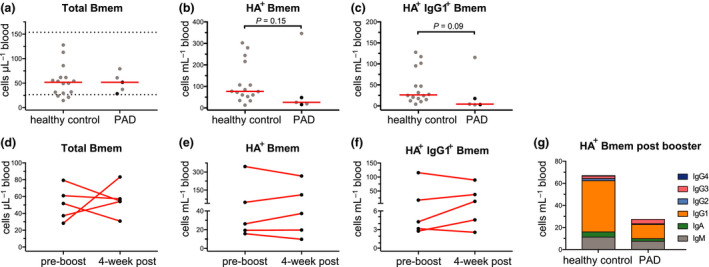
Detectable AM15 HA‐specific memory B cells in PAD patients. Absolute numbers of **(a)** total Bmem cells, **(b)** HA‐specific (HA^+^) Bmem cells and **(c)** HA^+^ IgG1^+^ Bmem cells in controls and PAD patients. Horizontal dotted lines represent the 5th and 95th percentiles of the control group and were used as reference ranges.^6^ Horizontal solid lines represent median values. Black dots represent those patients sampled more than 4‐weeks after booster vaccination (Table 2). Absolute numbers of **(d)** total Bmem cells**, (e)** HA^+^ Bmem cells and **(f)** HA^+^ IgG1^+^ Bmem cells in PAD patients pre‐ and > 4 weeks post‐booster vaccination. **(g)** Median values of absolute numbers of HA^+^ Bmem cells in controls and PAD patients 4‐week post‐booster vaccination. For gating strategy and population definitions, see Supplementary figure 1. Statistics were performed with the Wilcoxon matched‐pairs signed rank test for pre‐ and post‐booster vaccination and the Mann‐Whitney *U*‐test for unpaired groups between healthy control and PAD patients. * *P* < 0.05, ** *P* < 0.01.

All patients had normal numbers of total Bmem cells before booster vaccination (Figure 4a). PAD patients trended to have fewer total and IgG1^+^ HA‐specific Bmem cells than controls pre‐booster vaccination (*P* = 0.15; Figure 4b and c). Post‐booster vaccination samples were taken with a median of 29 days (range 29–62; Table 2). Following vaccination, no consistent changes were observed in the numbers of total Bmem cells (Figure 4d), HA‐specific Bmem cells (Figure 4e) or HA‐specific IgG1^+^ Bmem cells (Figure 4f). When compared to healthy controls, IgG3‐expressing Bmem cells trended to be higher in absolute numbers in PAD patients (Figure 4g) and were significantly higher as a proportion of total HA‐specific IgG^+^ Bmem cells than in healthy controls (*P* = 0.011; 25% vs 7.5% pre‐booster; Supplementary figure 3).

## Discussion

Herein, we show, by using fluorescently labelled antigen tetramers, that influenza‐specific Bmem cells are detectable 1 year after vaccination, mainly consist of IgG1^+^ cells, and these significantly expand post‐booster vaccination. Previously vaccinated PAD patients also carry HA‐specific Bmem cells, which are predominantly IgG1^+^. However, this cell population is smaller than in controls, and it does not expand following booster vaccination. Additionally, it contains a significantly higher proportion of IgG3^+^ Bmem cells.

For our studies, we devised an extensive flow cytometry stain that included mAbs to 13 cell surface markers and two fluorochrome‐conjugated HA‐antigen tetramers. Inclusion of mAbs to detect all major Ig isotypes and IgG subclasses allowed for unprecedented analysis of the vaccine antigen‐specific Bmem compartment. With this approach, we were able to recapitulate results from other studies regarding the expansion of Bmem following vaccination.^35^, ^49^, ^50^, ^51^, ^52^, ^53^, ^54^ Importantly, we expanded on this by showing a predominant IgG1 response. The availability of BUV dyes facilitated the design of a large flow cytometry panel. Moreover, these polymers are not protein‐based and therefore showed fewer B cells that specifically bound to one tetramer reagent rather than the protein fluorochromes PE and APC (unpublished observations).^56^, ^57^, ^58^

Our aim was to examine the impact of a booster vaccination on the resting Bmem cell compartment. We performed the post‐boost analysis after 1–2 months after vaccination. In previous studies, it has been determined that vaccination responses lead to expansions of plasmablasts after ~ 7 days and of activated Bmem cells at about 2 weeks. Both expansions were shown to be abrogated after 4 weeks.^35^ Thus, we conclude that the cells we examined post‐boost are those remaining after the initial expansion and more reflective of resting long‐lived Bmem cells.

Notably, in all individuals tested, circulating HA‐specific Bmem cells and neutralising antibodies were detected ~ 1 year after the 2018 vaccination at unexpectedly high levels. Although we cannot exclude the theoretical possibility that some individuals may have been exposed to a H1N1 strain and these cells were generated by natural infection rather than vaccination, this demonstrates that long‐lived antigen‐specific Bmem cells can be detected in blood.

Influenza‐specific Bmem cells in healthy controls have been previously identified between 14 and 28 days post‐vaccination.^35^, ^49^, ^50^, ^51^, ^52^, ^53^, ^54^ The immunophenotype of these cells was minimally explored but shown to be either CD27^+^,^49^, ^50^, ^54^ IgD^−^ ^50^, ^52^ or CD27^+^IgG^+^.^49^, ^51^ Our analysis agrees with these observations and further extends them through application of an extensive immunophenotyping panel including three Ig isotypes and four IgG subclasses. In healthy controls, the vast majority of HA‐specific Bmem cells are IgG1^+^ both before and after booster vaccination. Furthermore, the majority are CD27^+^ and this fraction was increased following booster vaccination. Previously, IgG1 was shown to be the predominant IgG subclass produced in serum in healthy controls post‐influenza vaccination.^59^, ^60^ IgG1 is important for complement fixation and viral neutralisation.^48^, ^61^, ^62^ However, it is unknown whether a predominant IgG1 Bmem cell response is desirable for influenza vaccination and whether it provides the best protection.

In contrast to the HA‐specific Bmem compartment, within the total CD27^+^IgG^+^ Bmem cell population, there is a large IgG2^+^ subset of about 20–25%. This is in line with our previous observations that in adults, the proportion of IgG2^+^ Bmem cells increases and that these are predominantly CD27^+^, contain high levels of SHM and contain molecular signs of consecutive Ig CSR.^31^, ^34^ It remains unclear as to why the HA‐specific Bmem cells following booster retained the IgG1 isotype. Potentially, the vaccine formulation skews the response to IgG1 and there is no consecutive Ig CSR. Alternatively, the vaccine induces only a primary GC response and there is a limited role for pre‐existing Bmem in secondary GC responses. This could potentially be addressed through purification of HA‐specific Bmem cells and molecular analysis of SHM in Ig‐variable gene regions and/or cloning of antibodies to determine their affinity.

Despite the presence of detectable numbers of HA‐specific Bmem cells in PAD patients, these were lower than in healthy controls. While our observations are limited by the small number of patients included in the study, we show that PAD patients had an increased proportion of Bmem cells expressing IgG3, the subclass encoded by the most *IGHM*‐proximal constant gene region. This observed increase is not just because of fewer IgG1^+^ Bmem cells because the fractions expressing IgG2 or IgG4 were not increased. Thus, the increased IgG3 usage could indicate a maturation defect, for example Ig CSR to more *IGHM*‐distal constant genes.^20^, ^63^, ^64^, ^65^

Vaccination responses are used in the diagnostic work‐up of PAD patients.^37^, ^41^, ^42^, ^66^, ^67^, ^68^, ^69^ However, many PAD patients are started on IgRT directly after they are found to have reduced serum IgG. Thus, the presence of antigen‐specific IgG in IgRT products often interferes with this analysis.^44^, ^45^ Detection of antigen‐specific Bmem cells could be a means to overcome this limitation, because the cells are not transferred from the donor plasma. We here show for the first time that Bmem populations in PAD patients contain cells with specificities to a relevant vaccine antigen. This can be very relevant because CVID patients with normal Bmem cell numbers have been shown to have a relatively mild clinical phenotype with regard to bronchiectasis and enteropathy.^5^, ^23^ Although such Bmem cells are impaired in generating Ab responses, these may act as antigen presenting cells to functional T cells,^36^ thereby playing an important role, especially in viral immunity. Influenza vaccination was used in this study as it is an ideal model for PAD patients as respiratory infections cause large burden on patient health and morbidity^7^, ^23^ and vaccination rates are as high as 80% is some patient cohorts.^68^ Since we here showed that influenza‐specific Bmem cells are readily detectable, even 1 year after previous infection, their presence can be examined without the need for a recent vaccination to determine the capacity of a PAD patient to generate antigen‐specific Bmem cells.

Flow cytometric detection of antigen‐specific B cells would allow patients to be classified further based on the functionality of their Bmem cell compartment. The presence of antigen‐specific Bmem cells could stratify patients based on the capacity to mount a B‐cell response and generate antigen‐specific Bmem cells that are potentially capable of assisting in T‐cell immunity. Those PAD patients that do not have antigen‐specific Bmem cells might be more prone to complications arising from infections. Furthermore, the immunophenotype of influenza‐specific Bmem cells may facilitate a better understanding of the nature of the immune defect in PAD patients.

## Conclusion

We were able to identify HA‐specific Bmem cells pre‐ and post‐booster influenza vaccination in healthy controls and PAD patients. We showed that the predominant phenotype of HA‐specific memory is IgG1 in healthy controls and PAD patients. PAD patients have detectable HA‐specific Bmem cells but lower in number than healthy controls. It has not been determined whether an IgG1 Bmem cell response is a desirable outcome in influenza vaccination. This preliminary study lays the framework for a larger cohort study where this approach could be used in a panel of diagnostic tests that is employed to further classify PAD patients based on the presence and immunophenotype of antigen‐specific Bmem.

## Methods

### Participants

From January to September 2019, 16 adult healthy controls and five adult patients with a clinical diagnosis of PAD were enrolled in a low‐risk research study to examine their peripheral blood B‐cell subsets (projects: Alfred Health 109/15, Monash University CF15/771 – 2015‐0344 and Monash University project 2016‐0289) (Tables 1 and 2). Participants consented to the donation of 40 mL of blood pre‐influenza booster vaccination and 28‐day post‐vaccination booster. All patients and healthy controls consented to the collection of basic demographics (age, sex) and relevant medical information, especially clinical diagnosis and histories of infectious and non‐infectious complications (Tables 1 and 2). All patients and healthy controls received the 2019 Australian quadrivalent vaccine, which was composed of two type A strains: A/Michigan/45/2015 (H1N1)pdm09‐like and A/Switzerland/8060/2017 (H3N2)‐like and two type B strains: B/Phuket/3073/2013‐like and B/Colorado/06/2017‐like.^70^ The study was conducted according to the principles of the Declaration of Helsinki and was approved by local human research ethics committees.

### Sample processing

Following blood sampling, total leucocyte counts were determined with the Cell Dyn analyser (Abbott core laboratory, Abbott Park, IL), and absolute numbers of leucocyte subsets were determined (see flow cytometry section). From the remaining sample, peripheral blood mononuclear cells (PBMC) were isolated by Ficoll–Paque density centrifugation and cryopreserved at a cell density of 10 million cells mL^–1^ in 50% RPMI, 40% FCS and 10% DMSO in liquid nitrogen for later analysis of antigen‐specific B cells.

### Protein production and tetramerisation

AM15 HA protein containing the Y98F mutation to limit sialic acid binding was produced as previously described.^54^ The HA construct consisted of the extracellular domain of the HA protein with a native leader sequence fused to the N‐terminus and the trimeric FoldOn domain fused to the C‐terminus followed by the biotin ligase (BirA) AviTag target sequence and a 6His affinity tag (Figure 1a). The DNA construct was cloned into a pCR3 plasmid and produced and purified as described previously.^52^, ^54^ Briefly, 30 μg plasmid DNA purified by Maxiprep (Zymo Research, Irvine, CA) was transfected in a 25‐mL culture of 293F cells using the Expi293 Expression system (Thermo Fisher, Waltham, Mass). Supernatant was collected on day 5 post‐transfection and purified by application to a Talon NTA‐cobalt affinity column (Takara Bio, Kusatsu, Shiga, Japan) and subsequent elution with 200 mm Imidazole. Eluted protein was then dialysed against 10 mm Tris for 48 h at 4°C. Protein was biotinylated by incubating at room temperature overnight with 1/8 of final volume each of Biomix A (0.5 m Bicine‐HCl, pH 8.3) and Biomix B (100mM ATP, 100 mM MgOAc, 500 μm D‐biotin) followed by 2.5 μg of BirA enzyme per milligram of protein. Biotinylated protein was subsequently dialysed against 10 mM Tris for 36 h at 4°C. Biotinylated protein was stored at −80°C prior to use. Soluble biotinylated AM15 HA protein was tetramerised by the addition of either Brilliant Ultra Violet (BUV)395‐conjugated streptavidin or BUV737‐conjugated streptavidin (BD Biosciences, Franklin lakes, NJ) at a protein:streptavidin molar ratio of 4:1.

### Protein gel

20 μL of sample (supernatant or purified protein) was mixed with 4 μL of 6 × non‐reducing or reducing buffer (0.1 m Tris‐HCl, pH 6.8, 0.2% bromophenol blue and 20% glycerol, reducing buffer included 4% SDS and 50 mm DTT). Samples under reducing conditions were heated to 85°C for 10 min. 10 μL of ladder (1:1 mixture) of Precision plus protein standard (Unstained and All blue, both from Bio‐Rad, Hercules, CA), reduced or non‐reduced sample was loaded on a 4–15% Mini‐PROTEAN TGX Stain‐Fee gel (Bio‐Rad) and run for 30 min at 200 V. The gel was subsequently imaged on the Bio‐Rad ChemiDoc Touch imaging system (Bio‐Rad).

### ELISA

Plasma‐specific HA IgG was measured by ELISA. Briefly, EIA/RIA plates (Costar, St Louis, MO) were coated with HA antigen (2 μg mL^–1^), blocked with 2% BSA and then incubated with diluted plasma samples (1:50). HA‐specific IgG was detected by adding rabbit anti‐human IgG HRP (Dako, Glostrup, Denmark). ELISA plates were developed using TMB solution (Life Technologies, Carlsbad, CA), and the reaction was stopped with 1 m HCl. Absorbance (OD450 nm) was measured using a Multiskan Microplate Spectrophotometer (Thermo Fisher). Serial dilutions of recombinant human IgG in separate wells on the same plate were performed for quantification of specific IgG.

### Hemagglutination inhibition (HI) assay

The HI assay was performed as described previously^52^, ^71^ using the WHO collaborating centre for reference and research in influenza HI typing assay kit. Briefly, duplicate plasma samples were serially diluted 2‐fold from 1:5 to 1:2560 and subsequently incubated with 4 HA units per 25 μL for 1 h. Following incubation, 1% human O group red blood cells were added, and plates were incubated for an additional hour at room temperature before inhibition was measured. HI titres reported are the reciprocal dilution of plasma that completely inhibited hemagglutination.

### Flow cytometry

Absolute numbers of leucocyte subsets were determined as previously described.^6^ Briefly, 50 μL of whole blood was added to a Trucount tube (BD Biosciences) together with a 20 μL Ab cocktail containing antibodies to CD3, CD4, CD8, CD16, CD19, CD45 and CD56 (Supplementary tables 2 and 3) and incubated for 15 min at RT in the dark. 500 μL of 0.155 m NH_4_Cl was added and the sample incubated for 15 min at RT in the dark to lyse red blood cells. The tube was then stored in the dark at 4°C for up to 2 h prior to acquisition on the LSRII analyser or LSRFortessa X‐20 (BD Biosciences).

For tetramer staining, 12.5 million PBMC were incubated with fixable viability stain 700 (BD Biosciences), antibodies against CD3, CD19, CD21, CD27, CD38, IgA, IgG1, IgG2, IgG3, IgG4, IgM (Supplementary tables 2 and 3) and 5 μg mL^–1^ each of BUV395 and BUV737 AM15 HA tetramers for 15 min at room temperature in a total volume of 250 μL. In addition, 5 million PBMC were stained with fixable viability stain 700 (BD Biosciences), antibodies against CD3, CD19, CD27, CD38, IgM and IgD, and Streptavidin BUV395 and BUV737 (Supplementary tables 2 and 3). Following staining, cells were washed twice with FACS wash (0.1% sodium azide, 0.2% BSA in PBS) and filtered through a 70‐μm filter prior to acquisition on the BD LSRFortessa X‐20. Flow cytometer set‐up and calibration were performed using standardised EuroFlow SOPs, as previously described (Supplementary tables 4 and 5).^6^, ^72^

### Data analysis and statistics

All data were analysed with FlowJo v10 software (TreeStar, Ashland, Ore). Reference ranges for healthy controls were defined as being within the 5th and 95th percentiles of absolute cell numbers, as previously published.^6^ Statistical analysis using Prism 7 Software (GraphPad Software, La Jolla, CA) for matched pairs was performed with the non‐parametric Wilcoxon matched‐pairs signed rank test. Statistical analysis for unpaired groups was made with the non‐parametric Mann–Whitney *U*‐test. For all tests, *P* < 0.05 was considered significant.

## Author Contributions

**Gemma Hartley:** Formal analysis; Investigation; Methodology; Writing‐original draft. **Emily Edwards:** Conceptualization; Formal analysis; Investigation; Methodology; Writing‐review & editing. **Julian J Bosco:** Conceptualization; Data curation; Writing‐review & editing. **Samar Ojaimi:** Data curation; Project administration; Writing‐review & editing. **Rob Stirling:** Data curation; Project administration; Writing‐review & editing. **Paul Cameron:** Data curation; Project administration; Writing‐review & editing. **Katie Flanagan:** Funding acquisition; Writing‐review & editing. **Magdalena Plebanski:** Funding acquisition; Writing‐review & editing. **P Mark Hogarth:** Conceptualization; Methodology; Writing‐review & editing. **Robyn OHehir:** Conceptualization; Funding acquisition; Project administration; Writing‐review & editing. **Menno van Zelm:** Conceptualization; Funding acquisition; Data curation; Writing‐review & editing.

## CONFLICT OF INTEREST

The authors declare that no conflict of interest exists.
