# Supplementary material for: Influenza‐specific IgG1+ memory B‐cell numbers increase upon booster vaccination in healthy adults but not in patients with predominantly antibody deficiency
Source: Clin Transl Immunology. 2020 Oct 16;9(10):e1199. doi: 10.1002/cti2.1199 (PMC7563650; doi:10.1002/cti2.1199)
Supplement: Supplementary file 1 [file CTI2-9-e1199-s001.docx]

**Supplementary Tables (n= 5) and Figures (n=3)**

**Supplementary Table 1: HI titres pre- and post-booster vaccination**

| **Individuals** | **Age at booster (years)** | **Sex** | **Days post-vaccination sample** | **HI titre**  **pre-booster** | **HI titre**  **post booster** |
| --- | --- | --- | --- | --- | --- |
| **Controls** | | | | | |
| 1 | 39 | M | 31 | 640 | 640 |
| 2 | 29 | F | 33 | 640 | 640 |
| 3 | 35 | F | 31 | 1280 | 640 |
| 4 | 33 | F | 28 | 640 | 640 |
| 5 | 26 | F | 27 | 320 | 320 |
| 6 | 39 | F | 32 | 640 | 640 |
| 7 | 24 | M | 28 | 1280 | 640 |
| 8 | 25 | F | 31 | 1280 | 640 |
| 9 | 45 | F | 28 | 640 | 640 |
| 10 | 29 | F | 29 | 640 | 640 |
| 11 | 33 | M | 28 | 640 | 640 |
| 12 | 39 | F | 26 | 640 | 640 |
| 13 | 25 | M | 28 | 640 | 640 |
| 14 | 24 | M | 28 | 640 | 640 |
| 15 | 31 | F | 28 | 1280 | 640 |
| 16 | 43 | F | 32 | 640 | 640 |
| **PAD patients** | | | | | |
| 1 | 50 | F | 29 | 320 | 640 |
| 2 | 32 | F | 29 | 320 | 320 |
| 3 | 29 | M | 29 | 640 | 640 |
| 4 | 27 | F | 56-91 | 320 | ND |
| 5 | 49 | F | 62 | 640 | ND |
| ND, not determined | | | | | |

**Supplementary Table 2. Composition of the antibody panels**

|  | **fluorochrome** | | | | | | | | | | | | | | |
| --- | --- | --- | --- | --- | --- | --- | --- | --- | --- | --- | --- | --- | --- | --- | --- |
| tube | **BUV395** | **BUV737** | **BV421** | **BV510** | **BV711** | **BV785** | **FITC** | **PerCP-Cy5.5** | **PE** | **PE-Vio615** | **PC7/**  **PE-Cy7** | **APC** | **AF700** | **APC-Cy7 / APC-H7** |  |
| 1. TruCount | **-** | **-** | **-** | **-** | **-** | - | CD3 | CD45 | CD16 + CD56 | **-** | CD4 | CD19 | - | CD8A |  |
| 2. HA-specific Bmem | HA-AM15 | HA-AM15 | CD27 | IgM | CD21 | CD3 | IgG2 + IgG3 | IgD | IgG1 + IgG2 | IgA | CD19 | IgG4 | Viability | CD38 |  |
| 3. Streptavidin control | Streptavidin | Streptavidin | CD27 | - | - | - | CD3 | IgD | - | - | CD19 | - | Viability | - |  |
| HA-AM15, hemagglutinin from the A/Michigan/2015 (H1N1) influenza strain | | | | | | | | | | | | | | |  |

**Supplementary Table 3. Antibody list**

| **Marker** | **Fluorochrome** | **Clone** | **Source** | **Cat. number** | **volume/**  **test (in μL)** | **tube(s)** |
| --- | --- | --- | --- | --- | --- | --- |
| CD3 | FITC | UCHT1 | BD Biosciences | 555332 | 3, 1 | 1, 3 |
| CD3 | BV785 | UCHT1 | BD Biosciences | 563918 | 1 | 2 |
| CD4 | PC7 | SFCI12T4D11 | Beckman Coulter | 6607101 | 0.2 | 1 |
| CD8A | APC-H7 | SK1 | BD Biosciences | 560179 | 4 | 1 |
| CD16 | PE | B73.1 | Biolegend | 360704 | 0.2 | 1 |
| CD19 | APC | SJ25C1 | Biolegend | 363006 | 0.4 | 1 |
| CD19 | PE-CY7 | SJ25C1 | BD Biosciences | 557835 | 5 | 2 |
| CD21 | BV711 | B-ly4 | BD Biosciences | 563163 | 5 | 2 |
| CD27 | BV421 | M-T271 | BD Biosciences | 562513 | 1 | 2, 3 |
| CD38 | APC-Cy7 | HIT2 | Biolegend | 303534 | 0.2 | 2 |
| CD45 | PerCP-Cy5.5 | 2D1 | BD Biosciences | 340953 | 2 | 1 |
| CD56 | PE | B159 | BD Biosciences | 555516 | 5 | 1 |
| IgA | PE-Vio615 | REA1014 | Miltenyi Biotec | 130-116-882 | 1.5 | 2 |
| IgD | PerCP-Cy5.5 | IA6-2 | Biolegend | 348208 | 1.5 | 2, 3 |
| IgG1 | PE | SAG1 | Cytognos | CYT-IGG1PE | 1 | 2 |
| IgG2 | FITC | SAG2 | Cytognos | CYT-IGG2F | 2 | 2 |
| IgG2 | PE | SAG2 | Cytognos | CYT-IGG2PE | 2 | 2 |
| IgG3 | FITC | SAG3 | Cytognos | CYT-IGG3F | 2 | 2 |
| IgG4 | APC | SAG4 | Cytognos | CYT-IGG4AP | 2 | 2 |
| IgM | BV510 | MHM-88 | Biolegend | 314522 | 1 | 2, 3 |
| Streptavidin | BUV395 | - | BD Biosciences | 564176 | 8.78 | 3 |
| Streptavidin | BUV737 | - | BD Biosciences | 564293 | 8.78 | 3 |
| Viability | AF700 | - | BD Biosciences | 564997 | 0.1 | 2, 3 |

**Supplementary Table 4. Flow cytometer set-up.**

| **LSRFortessa X-20** | | **Fluorochromes used in this study** |
| --- | --- | --- |
| **355 nm** | |  |
| 379/28 | No LP | BUV395 |
| 525/50 | 505 LP | **-** |
| 740/35 | 690 LP | BUV737 |
| **405 nm** | |  |
| 450/50 | No LP | BV421 |
| 525/50 | 505 LP | BV510 |
| - | - | - |
| 610/20 | 600 LP | - |
| 670/30 | 635 LP | - |
| 710/50 | 685 LP | BV711 |
| 780/60 | 750 LP | BV785 |
| **488 nm** |  |  |
| 488/10 | No LP | SSC |
| 530/30 | 505 LP | FITC |
| 710/50 | 685 LP | PerCP-Cy5.5 |
| **561 nm** |  |  |
| 586/15 | No LP | PE |
| 610/20 | 600 LP | PE-Vio615 |
| 675/50 | 635 LP | - |
| 780/60 | 750 LP | PE-Cy7, PC7 |
| **640 nm** |  |  |
| 670/30 | No LP | APC |
| 730/45 | 690 LP | Fixable Viability Stain 700 |
| 780/60 | 750 LP | APC-H7, APC-Cy7 |

**Supplementary Table 5. Target values for 7^th^ peak of rainbow beads in fluorescent channels**

| **Fluorochrome** | **Channel** | Lower (-15%) | **Target MFI** | Upper (+15%) | recommendation |
| --- | --- | --- | --- | --- | --- |
| **BUV395** | **BUV395** | 17,000 | **20,000** | 23,000 | In-house |
| **BUV737** | **BUV737** | 21,2500 | **25,000** | 28,750 | In-house |
| **BV421** | **V450** | 100,452 | **118,178** | 135,905 | EuroFlow |
| **BV510** | **V525** | 93,871 | **110,436** | 127,002 | EuroFlow |
| **BV605** | **V610** | 47,731 | **56,154** | 64,577 | In-house |
| **BV711** | **V710** | 15,079 | **17,740** | 20,401 | In-house |
| **FITC, BB515** | **B530** | 28,752 | **33,826** | 38,900 | EuroFlow |
| **PerCP-Cy5.5** | **B710** | 66,846 | **78,642** | 90,438 | EuroFlow |
| **PE** | **YG586** | 32,381 | **38,095** | 43,809 | EuroFlow |
| **PE-Vio615** | **YG610** | 178,500 | **210,000** | 241,500 | In-house |
| **PE-Cy7** | **YG780** | 8,316 | **9,783** | 11,250 | EuroFlow |
| **APC** | **R670** | 158,639 | **186,634** | 214,629 | EuroFlow |
| **AF700** | **R730** | 121,550 | **143,000** | 164,450 | In-house |
| **APC-H7** | **R780** | 64,194 | **75,522** | 86,850 | EuroFlow |
| Spherotech Rainbow Calibration particles (8 peaks) 3.41µm; cat nr. RCP-30-5A, Lot No. EAG01  EuroFlow recommendation based on Kalina *et al.*^70^ | | | | | |

**Supplementary Figures (n=3)**





**Supplementary Figure 1. Gating strategy for HA-specific Bmem. (A)** Gating strategy to define CD19^+^ B cells and HA-specific (HA^+^) B cells. (**B)** Strategy to delineate CD19^+^ B cells into naive (CD27^+^ IgM^+^) and IgM^+^, IgG1^+^, IgG2^+^, IgG3^+^, IgG4^+^ and IgA^+^ B memory (Bmem). (**C)** Strategy to delineate HA^+^ B cells into naive (CD27^+^ IgM^+^) and IgM^+^, IgG1^+^, IgG2^+^, IgG3^+^, IgG4^+^ and IgA^+^ Bmem.

**
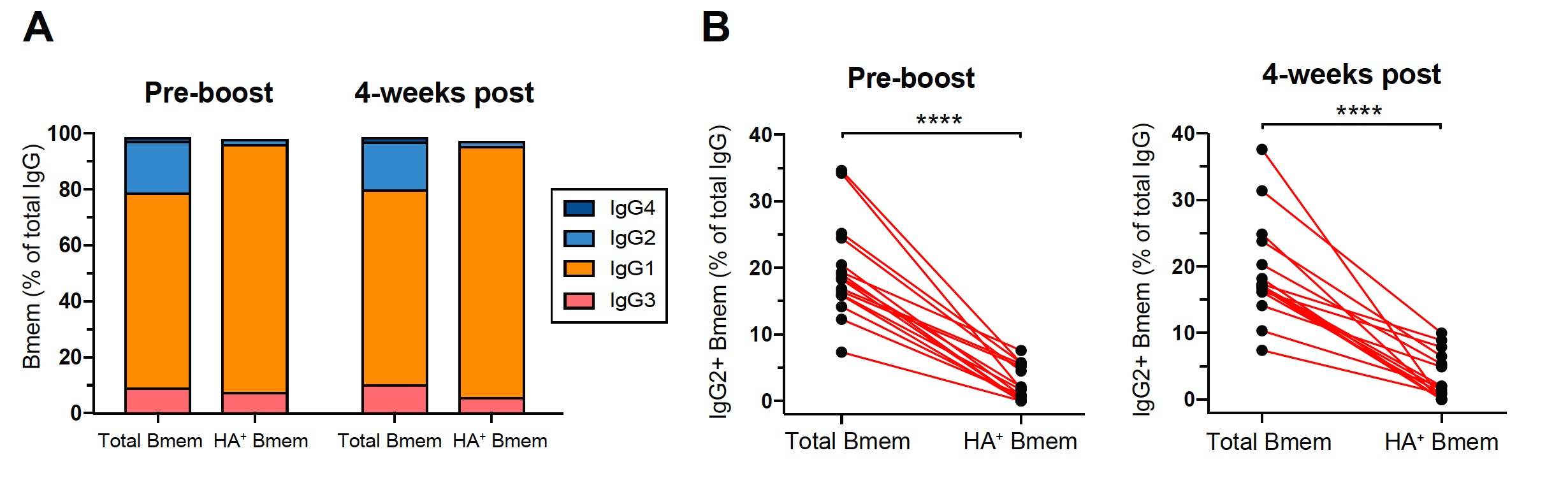
**

**Supplementary Figure 2. Total and HA-specific Bmem cells in healthy controls**

**(A)** Proportions of total and HA-specific (HA^+^) IgG1, IgG2, IgG3 and IgG4 memory B (Bmem) cells within total IgG Bmem cells. (**B)** Proportion of IgG2^+^ total and HA^+^ Bmem cells pre- and post-booster vaccination. Statistics were performed with the Wilcoxon matched pairs signed rank test for paired samples. **** *P* < 0.0001.


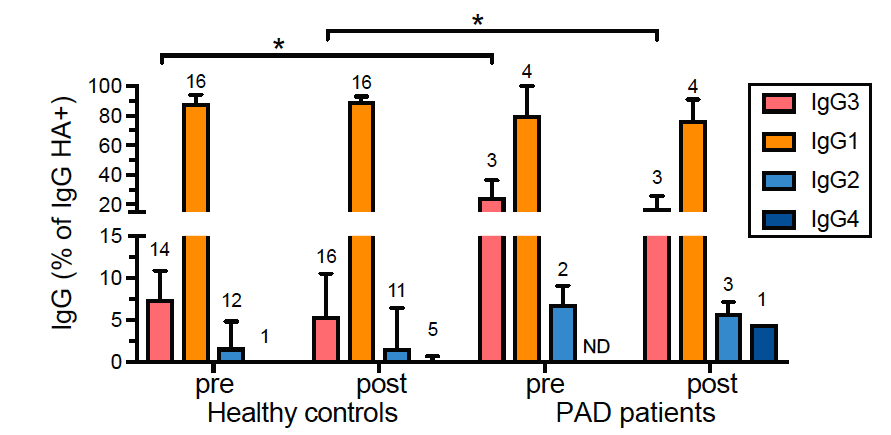


**Supplementary Figure 3. HA-specific IgG Bmem in PAD patients**

Proportions of HA-specific (HA^+^) IgG1^+^, IgG2^+^, IgG3^+^ and IgG4^+^ B memory within HA^+^ IgG^+^ B memory population in healthy controls and PAD patients. Numbers above each bar represent *n* numbers for each group. ND means not detected. Statistics were performed with the Wilcoxon matched-pairs signed rank test for pre- and post-booster vaccination and the Mann-Whitney test for unpaired groups between healthy control and PAD patients. * *P* < 0.05.
